# A Review of the Physiological and Immunological Functions of Biliary Epithelial Cells: Targets for Primary Biliary Cirrhosis, Primary Sclerosing Cholangitis and Drug-induced Ductopenias

**DOI:** 10.1080/17402520400004177

**Published:** 2004

**Authors:** Chih-Te Wu, Paul A. Davis, VelImir A. Luketic, M. Eric Gershwin

**Affiliations:** 1 Division of Rheumatology Allergy and Clinical Immunology University of California Davis School of Medicine Davis CA 95616 USA; 2 Division of Hepatology Medical College of Virginia Richmond VA 23298 USA

## Abstract

Our understanding of biliary epithelial cells (BEC) in physiobiology and immunology has steadily expanded. BEC transports IgA as well as IgM into bile, synthesizes and secretes various chemokines, cytokines, and expresses adhesion molecules involved in cell interaction and signal transduction. These then suggest a myriad of potential roles for BEC in defense from invading microorganisms as well as the pathogenesis of diverse immunologically driven diseases such as primary biliary cirrhosis (PBC), graft-versus-host disease, and primary sclerosing cholangitis (PSC). Despite the progress, there still remain many areas of BEC biology that require further investigation. Most importantly, it remains to be clarified that the extent to which the immunologic activities observed in BEC represent a BEC response to tissue injury or whether BEC themselves are the active participants in the pathogenesis of various cholestatic immunological diseases, including PBC and PSC.


*Clinical & Developmental Immunology*, September/December 2004, Vol. 11 (3/4), pp. 205–213

Taylor & Francis healthsciences

### A Review of the Physiological and Immunological Functions of Biliary Epithelial Cells: Targets for Primary Biliary Cirrhosis, Primary Sclerosing Cholangitis and Drug-induced Ductopenias

CHIH-TE WU[a,*], PAUL A. DAVIS[a,*,†], VELIMIR A. LUKETIC[b] and M. ERIC GERSHWIN[a]

[a]*Division of Rheumatology, Allergy and Clinical Immunology, University of California, Davis School of Medicine, Davis, CA 95616, USA;* [b]*Division of Hepatology, Medical College of Virginia, Richmond, VA 23298, USA*

Our understanding of biliary epithelial cells (BEC) in physiobiology and immunology has steadily expanded. BEC transports IgA as well as IgM into bile, synthesizes and secretes various chemokines, cytokines, and expresses adhesion molecules involved in cell interaction and signal transduction. These then suggest a myriad of potential roles for BEC in defense from invading microorganisms as well as the pathogenesis of diverse immunologically driven diseases such as primary biliary cirrhosis (PBC), graft-versus-host disease, and primary sclerosing cholangitis (PSC). Despite the progress, there still remain many areas of BEC biology that require further investigation. Most importantly, it remains to be clarified that the extent to which the immunologic activities observed in BEC represent a BEC response to tissue injury or whether BEC themselves are the active participants in the pathogenesis of various cholestatic immunological diseases, including PBC and PSC.

*Keywords*: Biliary epithelial cells; Primary biliary cirrhosis; Primary sclerosing cholangitis; IgA

### INTRODUCTION

The liver is composed of multiple cell types including two types of epithelia: hepatocytes, the major epithelial cells and intrahepatic biliary epithelial cells (BEC), which constitute 3–5% of the nuclear population of the liver (Tavoloni, 1987). BEC are typical epithelia, forming an extensive interconnecting network of intrahepatic and extrahepatic conduits which are heterogeneous with regard to diameter as well as their function. In humans, intrahepatic bile ducts range in size from 800 μm hepatic ducts to $<15\,\mu m$ bile ductules. Physiologically, BEC participate in a variety of fundamental processes including formation of bile as well as the transport of IgA into bile. In recent years, it has become apparent that BEC represent critical targets for a diverse group of diseases, including primary biliary cirrhosis (PBC), primary sclerosing cholangitis (PSC) as well as drug-induced ductopenias. In this review, we will focus on the physiological as well as immunological functions of the BEC.

### BILE FORMATION AND ELECTROLYTE TRANSPORT

Bile formation requires both hepatocytes and BEC as primary bile is extensively modified by BEC after its secretion by hepatocytes and prior to its delivery to the duodenum. BEC are responsible for approximately 30% of bile volume, a percentage that can be promptly increased to meet the changing physiological demands (Nathanson and Boyer, 1991). BEC secrete fluid, bicarbonate, chloride and immunoglobulin (Ig)A or reabsorb glucose, bile acids, amino acids, and electrolytes (Strazzabosco, 1997). The electrolyte transport function of the BEC is finely regulated by a complex system of intestinal hormones, neuropeptides, and neurotransmitters that promote either secretion or absorption. For example, secretin, which will be discussed in detail later, can increase the hydration and alkalinity of bile by stimulation of BEC secretion of chloride and bicarbonate.

### BEC SECRETORY PROCESSES

Secretion into the bile is clearly a major function of BEC as they are a major determinant of alkalinity and hydration of bile and contribute significantly to the local bicarbonate needed for digestion. BEC bicarbonate secretion is driven by the apically located $Cl^-/HCO_3^-$ exchanger, which is functionally coupled with the Cystic Fibrosis Transmembrane Conductance Regulator (CFTR), a cyclic AMP

*Contributed equally to manuscript.
†Corresponding author. Address: Division of Rheumatology, Allergy and Clinical Immunology, TB 192, University of California at Davis School of Medicine, Davis, CA 95616, USA. Tel.: +1-530-752-2884. Fax: +1-530-754-6047. E-mail: padavis@ucdavis.edu

ISSN 1740-2522 print/ISSN 1740-2530 online © 2004 Taylor & Francis Ltd
DOI: 10.1080/17402520400004177

(cAMP)-dependent $Cl^-$ channel. As noted earlier, secretin plays a major role in bicarbonate secretion as upon binding to its receptor, it increases intracellular cAMP levels, probably through a Gi-protein mediated induction of adenylate cyclase (Alvaro *et al.*, 1997a). Once the intracellular cAMP increases, cAMP-dependent protein kinase A (PKA) is activated and phosphorylates thereby activating the regulatory domain of CFTR, a critical step before the stimulation of bicarbonate secretion (Alvaro *et al.*, 1993; 1997b). This produces an open $Cl^-$ conductance (Frizzel and Morris, 1994) and results in BEC mediated secretion of $Cl^-$ into the bile. The BEC driven rise in bile $Cl^-$ increases the $Cl^-/HCO_3^-$ exchanger activity, leading to increased $HCO_3^-$ secretion into the bile (in exchange of $Cl^-$ absorption into the BEC). Interestingly, the apical $Cl^-/HCO_3^-$ exchanger lacks the ability to be stimulated by cAMP and therefore association with CFTR is required for cAMP-dependent $HCO_3^-$ secretion. This $Cl^-/HCO_3^-$ exchanger has been localized to the canalicular membrane of hepatocytes as well as the apical membrane of small and medium-sized bile ducts (Martinez-Anso *et al.*, 1994) using monoclonal antibodies and to the medium and large bile ducts along with the secretin receptor and CFTR (Alpini *et al.*, 1996) using functional studies.

Ductal bicarbonate secretion is also stimulated by bombesin and vasoactive intestinal peptide (VIP). Both of them, like secretin, enhance the activity of the $Cl^-/HCO_3^-$ exchanger after CFTR activation (Cho *et al.*, 1997a,b). Conversely, ductal bicarbonate secretion is inhibited by somatostatin and gastrin. Somatostatin, after binding to SSTR2 subtype receptors, which are detected almost exclusively on large bile ducts, inhibits basal and secretin-stimulated ductal bile flow. Somatostatin can also inhibit the secretin-induced increase of intracellular cAMP levels via a Gi-protein mediated inhibition of adenylate cyclase activity (Tietz *et al.*, 1995). Gastrin also inhibits secretin-induced bicarbonate secretion by decreasing the secretin-induced cAMP levels and the expression of secretin receptor in BEC (Glaser *et al.*, 1997). In contrast to somatostatin, gastrin does not affect basal bile flow.

Neural pathways also modulate $HCO_3^-$ secretion as vagal (cholinergic) stimulation increases $HCO_3^-$ secretion after acetylcholine binding to $M_3$ muscarinic receptors on the basolateral membrane of BEC. $M_3$ receptor activation increases intracellular $Ca^{2+}$ which recruits calcineurin, a $Ca^{2+}$- and calmodulin-dependent serine/threonine protein phosphatase 2B, that in turn sensitizes specific adenylate cyclase isoforms to secretin and thereby doubles intracellular cAMP. This finally results in an almost maximal stimulation of the $Cl^-/HCO_3^-$ exchanger (Alvaro *et al.*, 1997c; Alvaro, 1999). This effect is blocked by $M_3$ muscarinic receptor antagonists, intracellular $Ca^{2+}$ chelators, as well as the calcineurin inhibitors, FK-506 and cyclosporin A (Alvaro *et al.*, 1997c). The secretory processes of BEC are also regulated by ATP. ATP increases intracellular $Ca^{2+}$, induces intracellular alkalinization (activation of $Na^+/H^+$ exchanger) and, as a consequence, enhances the activity of the $Cl^-/HCO_3^-$ exchanger (Elsing *et al.*, 1996; Fitz, 1997; Strazzabosco, 1997). The coordinated regulation of bicarbonate secretion by secretin (cAMP) and acetylcholine ($Ca^{2+}$) could serve as a means of amplifying the secretory response just when the bicarbonate requirement in the intestine is maximal, i.e. the parasympathetic predominant digestive phase.

A number of different ion channels and carriers, in addition to the $Cl^-/HCO_3^-$ exchanger and CFTR, have also been identified on BEC. On the basolateral membrane, there are the $Na^+/H^+$ exchanger isoform 1 (NHE1), the $Na^+$-dependent $Cl^-/HCO_3^-$ exchanger, the $Na^+$–$K^+$–ATPase, and the $Na^+/K^+/2Cl^-$ cotransporter. The $Na^+$-dependent $Cl^-/HCO_3^-$ exchanger absorbs $HCO_3^-$ into the cell (Strazzabosco *et al.*, 1997). The $Na^+$–$K^+$–ATPase maintains the $Na^+$ gradient and, together with $K^+$ channels, determines the membrane potential difference of BEC. The $Na^+/K^+/2Cl^-$ cotransporter actively absorbs $Cl^-$ ions into the cell and appears to be a major determinant of fluid secretion (Singh *et al.*, 1996). On the luminal surface of BEC, a $Ca^{++}$-activated $Cl^-$ channel is present which may be activated by luminal purinergic nucleotides (Strazzabosco *et al.*, 2000). More recent work has demonstrated the expression of the apical isoforms of $Na^+/H^+$ exchanger (NHE2) (Spirli *et al.*, 1998), which enhance $Na^+$ reabsorption. Figure 1 summarizes the different electrolyte transport mechanisms in BEC.

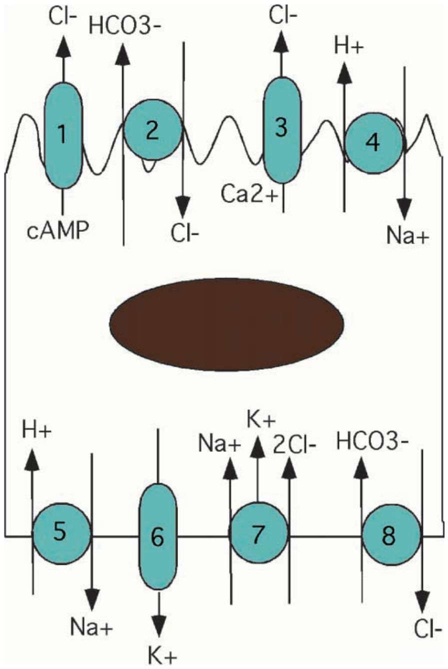

FIGURE 1 Mechanisms of electrolyte transport in biliary epithelial cells. At the apical membrane, the cystic fibrosis transmembrane conductance regulator (CFTR) (1) is couplewith the $Cl^-/HCO_3^-$ exchanger (2), which secretes $HCO_3^-$ in exchange of $Cl^-$ absorption. There is another $Ca^{2+}$-dependent $Cl^-$ channel (3) as well as the $Na^+/H^+$ exchanger isoform 2 (NHE2) (4). On the basolateral side of the BEC, there is $Na^+/H^+$ exchanger isoform 1 (NHE1) (5) and $K^+$ channel (6). Another type of $Cl^-/HCO_3^-$ exchanger (7) and a $Na^+/K^+/2Cl^-$ exchanger are also present.

### ABSORPTIVE FUNCTIONS

BEC are also capable of active reabsorption of glucose, bile acids, glutamate, conjugated bilirubin, sulfobromophthalein, and low molecular weight organic anions which further modify the final constituents of bile.

In isolated perfused rat livers, BEC absorbs both metabolizable hexose (D-glucose) and pentose (D-xylose) as well as nonmetabilizable hexoses (α-methylglucoside, 3-*o*-methylglucose, and L-glucose) (Lira *et al.*, 1992). Because in sodium-free media, absorption of L-glucose and α-methylglucoside gradually ceased while absorption of D-glucose and 3-*o*-methylglucose remained unchanged, there are at least two different sugar transport systems present.

Active transport processes for conjugated bile acids have recently been described using isolated rat cholangiocytes (Lazaridis *et al.*, 1997). Conjugated bile acids were absorbed in a $Na^+$-dependent fashion at the luminal BEC surface. Furthermore, immunohistochemistry also demonstrated the apical localization of the rat ileal $Na^+$-dependent bile acid transporter (ASBT). The meaning of the reabsorption remains to be clarified. However, intracellular bile acids are capable of modulating many important intracellular events including the level of secondary messengers ($Ca^{2+}$, cAMP), vesicle trafficking, ion transport, growth and replication (Alvaro, 1999). Unlike conjugated bile acids, lipophilic, unconjugated bile acids, such as ursodeoxycholic acids, are passively reabsorbed. This provides the first essential step in the cholehepatic shunt model (Hofmann *et al.*, 1997), which explains the phenomenon of bicarbonate rich hypercholeresis generated by these bile acids. The same phenomenon was also demonstrated for weakly acidic drugs, such as the anti-inflammatory sulindac (Hofmann *et al.*, 1997). This has lead to speculation that cholehepatic recycling of the substances reabsorbed by BEC represents an additional removal step for unconjugated substances from the bile. As a major function of the liver is metabolism and conjugation of xenobiotics and drugs to be then secreted mainly as water-soluble metabolites into bile, substances that escape hepatic conjugation/metabolism might be excreted as hydrophobic (toxic) substances. However passive reabsorption by BEC and the continuous recycling likely are designed to ensure further conjugation/metabolism of these toxic metabolites. Final biliary excretion appears to occur only when this class of substances is rendered hydrophilic and non-toxic. In parallel, bile acids are also efficiently recycled under normal conditions after their release. Unconjugated, and to a lesser extent also conjugated, bile acids are absorbed by passive diffusion throughout the entire gut. However, an active transport mechanism for conjugated bile acids exists in the distal ileum. The reabsorbed bile acids enter the portal circulation and are taken up rapidly by hepatocytes, metabolized, and resecreted into the bile (enterohepatic circulation). Normally, the bile acid pool circulates approximately 5–10 times daily and intestinal reabsorption is 95% efficient, so fecal loss of bile acids is minimal. This loss is compensated by an equal daily synthesis of bile acids by the liver, thereby the size of the bile acid pool remains unchanged.

L-glutamate is released within the biliary tree from glutathione via the action of γ-glutamyl transferase (GGT). It has been shown that BEC possess the capacity for a sodium-dependent and sodium-independent transport system for L-glutamate. These transport systems are different from the hepatocellular system since the inhibition and kinetic properties are different (Eisenmann-Tappe *et al.*, 1991). However, the localization of these transporters has not been identified yet, and therefore the physiological role of such a transport system remains unclear.

### IGA SECRETION

IgA is the major protein in bile and is the major and characteristic immunoglobulin of the mucosal immune system (Lemaitre-Coelho *et al.*, 1977). Two different pathways of IgA transport into bile are present. In humans, the IgA is synthesized locally in plasma cells along the biliary tree, bound to polymerized immunoglobulin receptor (pIgR) on the basolateral membrane of BEC, and transported, and secreted into the bile by BEC. In rats, the IgA is cleared from plasma by pIgR located on the sinusoidal surface of hepatocytes and delivered into bile. The major pathway for IgA transport into bile is via a receptor-binding interaction (Fig. 2). Studies in rats have demonstrated that IgA is transported by pIgR, an extracellular receptor present in the sinusoidal membrane of hepatocytes. pIgR is synthesized as a transmembrane glycoprotein and is expressed in a polar fashion on the basolateral (or sinusoidal) surface of the cell, where it is available to bind IgA. pIgR is synthesized by the epithelial cells of all external-secreting organs (intestine, mammary gland, lung, lacrimal gland, endometrium, etc.) as well as by the hepatocytes and BEC (Takahashi *et al.*, 1982; Delacroix *et al.*, 1984; Daniels and Schmucker, 1987), as summarized in Table I.

IgA itself is synthesized in IgA-specific plasma cells as heavy chains (alpha) and light chains (kappa or lambda) which are assembled into monomeric IgA molecules (two heavy chains and two light chains). In the presence of the J chain addition peptide, monomeric units join via disulfide linkages to create dimers and other multimeric forms of IgA, which are then secreted. Only polymeric forms of IgA have high affinity for and thus can bind to pIgR. A tyrosine residue in the IgA molecule plays an important role in the binding of IgA to pIgR, since binding and transcytosis of IgA often are decreased after iodination of the IgA (Schiff *et al.*, 1986). The J chain is required for the binding of IgA to pIgR and subsequent intracellular transport of IgA (Brandtzaeg and Prydz, 1984), although pIgR does not interact directly with the J chain. The first homologous domain at the $NH_2$-terminal of pIgR

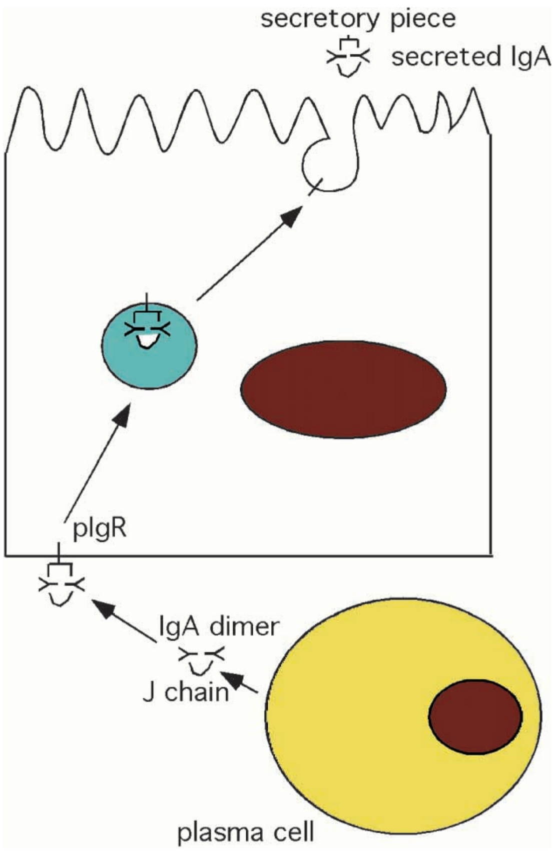

FIGURE 2 Schematic representation of the transcellular pathway for IgA secretion. Dimeric IgA secreted by plasma cells binds to pIgR, which is located on the basolateral membrane of BEC. The IgA–pIgR complex is internalized and transported across BEC is the vesicle. Upon arriving the apical pole of BEC, pIgR is proteolytically processed, which releases IgA coupled with secretory piece.

is necessary for binding IgA (Hanly *et al.*, 1987) and at the BEC surface, IgA first interact with pIgR noncovalently (Lindh and Bjork, 1976). Later, the free sulfhydryl group on the IgA molecule initiates the so-called disulfide interchange reaction by reducing a disulfide bond on the pIgR. This reaction leads ultimately to the formation of two new inter-molecular disulfide bonds between the IgA molecule and pIgR (Pardo *et al.*, 1979). Interestingly, even though some degree of cross-affinity between IgA and pIgR of different species exists, the ability of IgA from one species to bond covalently to pIgR of different species is quite variable suggesting that the reaction requires certain conformational characteristics of both the IgA and the pIgR (Socken and Underdown, 1978).

TABLE I Predominant cellular location of pIgR in hepatobiliary tissues in various animal species

| Species | Location of pIgR |
|---|---|
| Rat | Hepatocytes |
| Rabbit | Hepatocytes |
| Mouse | Hepatocytes |
| Human | BEC |
| Monkey | Unknown (not on hepatocytes) |
| Guinea pig | BEC |

After binding, the IgA–pIgR complexes are internalized into coated endocytic vesicles which are routed across the epithelial cells (transcytosis) to the apical pole. Transport of the formed complexes is size dependent as pentameric IgM and large IgA immune complexes are generally transported less efficiently into bile than dimeric IgA (Socken *et al.*, 1981). For example, complexes of trinitrophenylated bovine thyroglobulin (molecular weight of approximately 970 kDa) and polymeric IgA were transported less well than IgA-trinitrophenylated human albumin complexes (molecular weight of approximately 460 kDa). Soon after endocytosis, the IgA–pIgR complexes are localized in a vesicular–tubular network, where the sorting of IgA–pIgR complexes from other endocytosed ligands presumably occurs. The vesicles containing IgA–pIgR complexes, smooth-coated and vary in size from 100 to 160 nm in diameter, then are transported across the cell, avoiding interaction with lysosomes and Golgi complexes, and fuse with the bile canalicular membrane (Hoppe *et al.*, 1985). This process also depends on functioning microtubules since treatment with colchicine abolishes the transport of IgA (Mullock *et al.*, 1980) The transcellular migration also appears to involve only discrete vesicles, as there was no extensive connection between structures in the sinusoidal and bile canalicular regions (Hoppe *et al.*, 1985).

The final step of the IgA delivery pathway involves the proteolytic cleavage of the IgA–pIgR complexes at the bile canalicular pole of the hepatocytes (or apical pole of the BEC). At this step, pIgR is proteolytically processed to yield two proteins: a soluble fragment of pIgR called secretory piece (SC) and a transmembrane anchoring fragment containing the cytoplasmic tail of pIgR, which remains on the vesicular membrane. The SC is released into the bile alone with IgA, where it acts to protect the IgA from degradation thereby increasing the half-life of IgA in the bile. The exact mechanism and location of this proteolytic cleavage remain incompletely understood. Immunohistochemical studies have shown an accumulation of IgA and pIgR at or near the bile canalicular membrane before exocytosis of IgA (Takahashi *et al.*, 1982), suggesting that the fusion of the endocytic vesicle with the bile canalicular membrane or the proteolysis of pIgR is the rate-limiting step in the pathway. By using the monoclonal antibodies specific for the cytoplasmic tail of pIgR, it has been shown that either the cytoplasmic tail itself or other slightly degraded fragments are also released into the bile, as opposed to being degraded intracellularly (Solari *et al.*, 1986). Interestingly, the SC is proteolytically processed for a second time by a metalloprotease present on the brush border plasma membrane of the jejunum into a smaller form (Ahnen *et al.*, 1986).

The role and, therefore, the biological significance of the transport of IgA from the plasma into the bile have not been clearly defined. However, there are at least three proposed functions (Fig. 3). First, IgA in bile provides protection for the biliary tree and liver against invading pathogens by

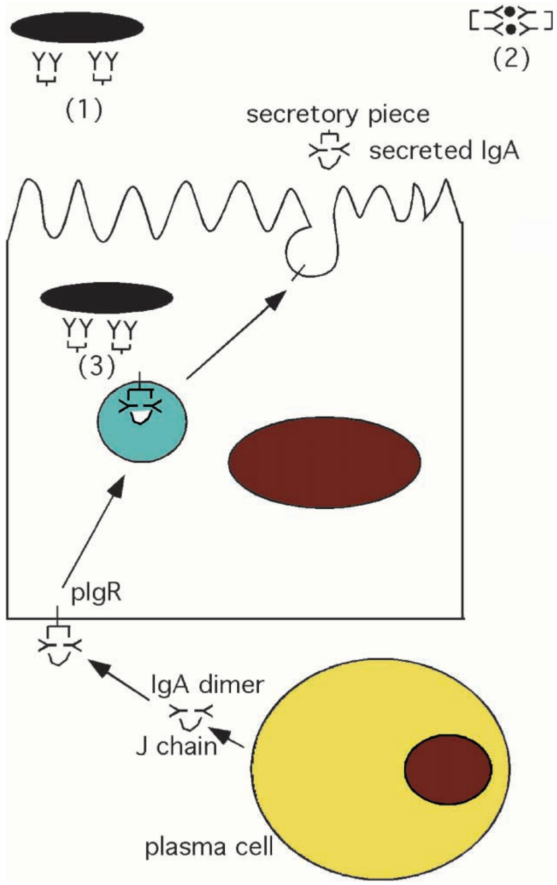

FIGURE 3 Proposed function of antibody in the bile. (1) Binding to extracellular pathogen and preventing its attachment to the BEC. (2) Binding to free antigen, forming immune complex, and facilitating its excretion. (3) Binding to intracellular pathogen during its transcytosis.

preventing the attachment of microorganisms and/or their toxins. Natural IgA antibodies to various intestinal bacteria are present in bile and inoculation into the intestinal lumen or intestinal lymphoid tissues of various antigens resulted in specific IgA antibodies in bile and protected experimental animals from, for example, cholangitis (Aagaard *et al.*, 1996). Second, potential harmful antigens present in the circulation can be cleared into bile in the form of immune complexes with IgA, thereby reducing the possibility of a systemic reaction. Indeed, transport of intravenously injected free antigen into bile by circulating IgA, either passively administered or actively induced, has been demonstrated in rats. This could be especially important since a state of chronic inflammation might constantly arise given that the gastrointestinal tract is in contact with enormous diversity and amount of antigens daily (Harmatz *et al.*, 1982; Peppard *et al.*, 1982). A third possible role for the transport of IgA from plasma into bile is to provide specific protection against invading microorganisms as during the transcytosis process, the IgA antibodies can encounter and bind to an intracellular microbial pathogen. For example, co-localization of viral envelope proteins of Sendai and influenza viruses and IgA monoclonal antibodies specific to that viral protein has been demonstrated by 2-color immunofluorescence in a recent study (Mazanec *et al.*, 1995).

Different animal species have different capacities to transfer IgA from plasma into bile as well as different location of pIgR within the liver. Other factors may also influence the IgA transport and pIgR expression. For example, the synthesis and expression of pIgR appears to be regulated during cellular differentiation and development. In the rat, immunorecognizable pIgR appears in intestinal epithelial cells between 10 and 15 days after birth, corresponding to the appearance in the lamina propria of IgA-producing plasma cells (Nagura *et al.*, 1978). pIgR then reaches adult levels of expression by 40 days after birth. In rats, the hepatobiliary secretion of IgA decreases with age, as a direct result of an age-related decrease in the number of pIgR present on hepatocytes (Daniels *et al.*, 1985). In contrast, in humans, pIgR has a different time course and is observed in fetal tissues prior to the appearance of either IgA or IgA-producing plasma cells (Ogra *et al.*, 1972).

The regulation of the expression of pIgR, and consequently the transport of IgA into external secretions may be tissue-specific as well as dependent on specific hormones and other chemical influences. The amount of pIgR synthesized by uterine or lacrimal cells may be influenced by sex steroids (Sullivan and Wira, 1981; Sullivan *et al.*, 1984) as pIgR concentration in the tears of male rats is approximately 5-fold greater than that in a female. Castration results in a decrease of pIgR concentration in the tears. In hepatocytes, regulation of pIgR synthesis by glucocorticoids was also noted (Wira and Colby, 1985) as pIgR increased significantly after the addition of cortisol to the culture medium. Furthermore, this effect was abolished when either estradiol or cyclohexamide was added to the medium, which indicates that expression of pIgR is controlled both transcriptionally and translationally. Additionally, the synthesis and secretion of pIgR by HT-29 cells, a human colonic carcinoma cell line, can be increased by treatment with $\gamma$-interferon ($\gamma$-IFN) (Sollid *et al.*, 1987), tumor necrosis factor (TNF)-$\alpha$ (Kvale *et al.*, 1988), or by growing the cells in glucose-free, galactose-substituted medium (Rao *et al.*, 1987).

The transport of IgA into bile is related to bile flow. Transient bile duct ligation (2 h) dramatically decreased liver transport of IgA in the period subsequent to ligation (Kloppel *et al.*, 1987). The amount of IgA secreted into the bile was decreased to one-tenth of control value before ligation. In the rat, treatment with 17$\alpha$-ethynylestradiol (5 mg/kg for 5 days) reduced bile flow by greater than 60%. In parallel, the transport of intact IgA into bile decreased by 43% (Goldsmith *et al.*, 1987). Table II summarizes the regulation of pIgR expression and/or IgA transport by various agents.

In addition to IgA, other subclasses of immunoglobulins are also present in the bile. The concentration of biliary IgG is less than 8% the concentration of serum IgG (Manning *et al.*, 1984). It appears that bile IgG mainly

TABLE II Regulation of pIgR expression/IgA transport by various agents

| Effect on pIgR expression/IgA transport | |
|---|---|
| Increase | Decrease |
| Androgen | Aging |
| Cortisol | Bile duct ligation |
| IFN-γ | 17α-ethynylestradiol |
| TNF-α | |

originates from diffusion from blood. However, this mechanism was not supported in one study on human bile after tetanus toxoid immunization. The kinetics of the biliary IgG response in this study differed significantly from that found in serum. Moreover, when compared with paired sera response, the peak response in the bile exceeded that of the sera and showed 19-fold more abundant IgG and 45-fold more abundant IgA than could have arrived by diffusion. This suggests that intrahepatic antibody production for export to the intestinal tract may occur in humans (Hansen *et al.*, 1989). Similar to the origins of IgG, trace amounts of IgM in bile may be either the result of leakage from blood or the presence of local IgM-producing plasma cells within the liver. The latter is supported by another study which showed that antigen entering the bloodstream stimulated a population of cells in the spleen to migrate to the liver. These cells then produce and secrete IgM into bile, thereby providing rapid and localized immunological response to invading microorganisms (Jackson and Walker, 1983).

### IMMUNE RESPONSE ROLE-SYNTHESIS OF CHEMOKINES, CYTOKINES, AND OTHER MEDIATORS

The ability of BEC to express and secrete chemokines and cytokines is crucial for biliary innate immune defenses since these cells are the initial contact points for potential pathogenic microorganisms in the bile or ascending from the intestinal tract via the biliary tree. Locally secreted chemokines and cytokines are important signals for recruitment of other immune cells. Human BEC express and secrete IL-8 and monocyte chemotactic protein-1 (MCP-1) promoting the recruitment of neutrophils and monocytes or T-lymphocytes, respectively, to the portal tracts. When stimulated with proinflammatory cytokines such as interleukin (IL)-1 or TNF-α, this response is rapidly upregulated. When BEC were cocultured in transwell chamber below monolayers of endothelial cells, the transendothelial migration of neutrophils was observed. This response was blocked by antibodies to CD18 or CD11 but only partially inhibited by antibodies to IL-8 (Morland *et al.*, 1997). IFN-γ had a differential effect since it reduced IL-8 but enhances MCP-1 secretion. The recruited neutrophils, monocytes, or T-lymphocytes can act as part of a protective response against ascending biliary infections or as participants in the pathological inflammation of the bile ducts like in PBC.

Studies of BEC in culture have shown that IL-6 synthesis and secretion into the medium. The TNF receptor and to a lesser extent IL-6 receptor α chain are also present in BEC. Damaged BEC also have an increased expression of both IL-6 mRNA and TNF-α mRNA compared to normal liver suggesting an possible autocrine effect (Yasoshima *et al.*, 1998). The secreted IL-6 and TNF-α could act in a paracrine or autocrine fashion as IL-6 increases BEC DNA labeling index from 4- to 6-fold after 24 h in primary cultures maintained in serum-free conditions and promotes BEC proliferation *in vitro* (Matsumoto *et al.*, 1994). Second, these cytokines have an effect on lymphocytes in the vicinity of portal tracts. For example, IL-6 may promote terminal differentiation of B cells and stimulate secretion of immunoglobulins. TNF-α may induce expression of various adhesion molecules on BEC (Ayres *et al.*, 1993) as well as the cytotoxic activities of T lymphocytes. Finally, IL-6 and TNF-α may mediate direct tissue damage by means of inflammation and apoptosis as treatment with TNF-α induced apoptosis in cultured rat hepatocytes (Bour *et al.*, 1996).

BEC also synthesize and secrete other peptides and mediators, such as transforming growth factor (TGF)-$\beta_2$ (Milani *et al.*, 1991), endothelin-1 (Caligiuri *et al.*, 1998), platelet-derived growth factor (PDGF)-B chain (Milani *et al.*, 1991), and nitric oxide (NO) (Vos *et al.*, 1997). BEC do not produce these mediators under normal physiological conditions, but actively synthesize them in many conditions of acute and chronic liver injury. Thus, activated BEC communicate extensively with other immunological cells, including lymphocytes, neutrophils, fibroblasts, and Kupffer cells (macrophages).

### IMMUNE RESPONSE ROLE-EXPRESSION OF ADHESION MOLECULES

BEC could intensify and localize the immune response by expressing selected cell surface adhesion molecules. Both *in vivo* and *in vitro* BEC from both diseased and normal livers express intercellular adhesion molecules (ICAM)-1, leukocyte function antigen (LFA) -3, major histocompatibility complexes (MHC)-I, and to a lesser degree, MHC-II constitutively in primary cultures (Ayres *et al.*, 1993; Cruickshank *et al.*, 1998) (Fig. 4). When treated with proinflammatory cytokines such as TNF-α, IFN-γ, or IL-1, a significant increase of ICAM-1, MHC-I, and MHC-II expression was noted from both the diseased and normal liver (Ayres *et al.*, 1993). In another study using BEC cell lines (PLC/PRF/5, HepG2, Hep3B, and CC-SW), all expressed MHC-I, LFA-3, ICAM-1, and EGF receptor. Furthermore, all but Hep3B expressed CD95. Similarly, proinflammatory cytokines such as IFN-γ and TNF-α upregulated surface expression of ICAM-1, MHC-1, and MHC-II. IL-4, a Th2 cytokine, also upregulated CD95

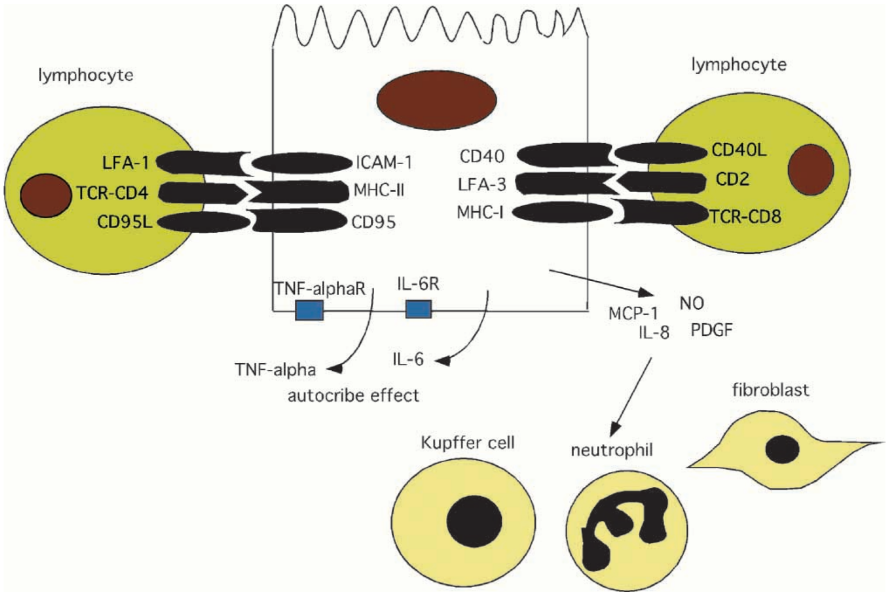

FIGURE 4 BEC constitutively express certain adhesion molecules, including ICAM-1, LFA-3, and CD40, which interact with surrounding lymphocytes expression LFA-1, CD2, and CD40L. BEC also express MHC-I and MHC-II, making them cytotoxic targets and/or becoming professional APC. Cytokines produced by BEC have either autocrine or paracrine effects, which can modulate the functions of other immunological cells in the vicinity.

expression while TGF-β, an anti-inflammatory cytokine, markedly downregulated cell surface expression of CD95, but increased expression of LFA-3 (Cruickshank *et al.*, 1998). The effect of proinflammatory cytokines on the expression of adhesion molecules and MHC-I is consistent with the hypothesis that BEC are not innocent bystanders during inflammatory responses but active participants in the inflammatory reactions observed in liver immune-mediated disorders (Table III).

As BEC express MHC-I and LFA-3, important adhesion molecules for lymphocyte trafficking and recognition, they can also act as targets for cytolytic T cells. It has been demonstrated that allogeneic lymphocytes could bind to human primary BEC cultures and this interaction was enhanced by activation of either participating cell types. At least 50% of the overall interaction is dependent on LFA-1 on cytotoxic T-lymphocytes, which is the ligand for ICAM-1 expressed on BEC. BEC are also susceptible to lysis by cytokine-activated natural killer (NK) cells (Leon *et al.*, 1997). In the presence of antigen in association with MHC molecules, the lymphocytes would become activated to recognize BEC as a potential target and

TABLE III Effect of cytokines on the expression of membrane surface markers in BEC

| | ICAM-1 | MHC-I | MHC-II | LFA-3 | CD95 | CD40 |
|---|---|---|---|---|---|---|
| Unstimulated | + | + | ± | + | + | + |
| TNF-α | ↑ | ↑ | ↑ | No effect | NA | ↑ |
| IFN-γ | ↑ | ↑ | ↑ | No effect | NA | ↑ |
| IL-1 | ↑ | ↑ | ↑ | NA | NA | NA |
| TGF-β | ↓ | ↓ | ↓ | ↑ | ↓ | No effect |
| IL-4 | NA | NA | NA | NA | ↑ | NA |

NA, not available.

expression of adhesion molecules would then render them susceptible to cytolytic mechanisms by attracting cytotoxic T lymphocytes (CTL). It is also possible that upregulated expression of MHC-I on BEC increases the avidity of the T cell receptor (TCR)/MHC interaction, which in turn increases their potential as target cells for T cell-mediated attack. The fact that LFA-3 is constitutively expressed on BEC suggests an interaction with CD2 on CTL and/or NK cells that may lead to cytotoxicity (Leon *et al.*, 1997). It has also been found that TCR stimulation or treatment with phorbol ester rapidly increased the avidity of CD2 for LFA-3 in murine T cell hybridomas transfected with human CD2 (Hahn *et al.*, 1993). Cell lines expressing single amino acid mutations of the carboxyl terminal asparagines of CD2 lost CD2 avidity regulation through TCR activation. This regulation requires both protein tyrosine kinases and protein kinase C. Agents that increase intracellular cAMP levels also upregulated CD2 avidity. In conclusion, the CD2/LFA-3 interaction not only acts as the physical bridge between T lymphocytes and BEC but also are affected by or turn on other signal transduction pathways

Viral infection can also modulate BEC expression of adhesion molecules. For example, cytomegalovirus (CMV) infection increased BEC surface expression of MHC-I but not MHC-II (Scholz *et al.*, 1997). This augmentation of MHC-I expression was observed when the virally infected BEC was cocultured with autologous but not allogeneic peripheral blood lymphocytes. CMV also reduced the IFN-γ mediated induction of MHC-II while MHC-I was unchanged. This also suggests that a viral infection can modulate the immunogenic potential of BEC by making them more susceptible to T lymphocyte attack due to increased MHC-I expression.

An earlier study has confirmed the constitutive expression of MHC-II from primary BEC cultures of both normal and diseased livers (Cruickshank *et al.*, 1998) and that proinflammatory cytokines such as TNF-α, IFN-γ, or IL-1 upregulated the MHC-II expression of BEC. The reason for this expression of MHC-II on BEC remains unclear although this has lead to suggestions that BEC might act as professional antigen presenting cells (APC) as APC are characterized by MHC-II expression. For example, it has been shown that the increased destruction of bile duct epithelium is associated with MHC-II-specific lymphocytes. *In vivo* studies have also shown that intraperitoneal administration of IL-2 in mice induces not only luminal expression of MHC-II on BEC, but also lymphocyte infiltration around bile ducts in liver (Himeno *et al.*, 1992). Most infiltrating lymphocytes were T cells and about one-third of them were positive for IFN-γ suggesting that the infiltration of lymphocytes is dependent on endogenous IFN-γ production. However it is well accepted that the expression of MHC-II alone is not sufficient for antigen presentation to naïve T lymphocytes as successful primary activation and subsequent proliferation of T cells by APC requires costimulatory signals, B7-1 (CD80) and B7-2 (CD86). However, neither CD80 nor CD86 are constitutively expressed by unstimulated human BEC and high concentrations of either IFN-γ, TNF-α, or both in combination failed to induce their expression on BEC (Leon *et al.*, 1997). While this argues that BEC are unlikely to act as professional APC, it is still possible that BEC may present antigens in an inefficient manner which may result in specific T cell anergy or deletion. The anergic T cells may function as suppressor cells, inhibiting subsequent T cell activation even in the presence of professional APC.

### References

Aagaard, B.D., Heyworth, M.F., Oesterle, A.L., *et al.* (1996) "Intestinal immunisation with *Escherichia coli* protects rats against *Escherichia coli* induced cholangitis", *Gut* **39**, 136–140.

Ahnen, D.J., Singleton, J.R., Hoops, T.C. and Kloppel, T.M. (1986) "Posttranslational processing of secretory component in the rat jejunum by a brush border metalloprotease", *J. Clin. Investig.* **77**, 1841–1848.

Alpini, G., Roberts, S., Kuntz, S.M., *et al.* (1996) "Morphological, molecular, and functional heterogeneity of cholangiocytes from normal rat liver", *Gastroenterology* **110**, 1636–1643.

Alvaro, D. (1999) "Biliary epithelium: a new chapter in cell biology", *Ital. J. Gastroenterol. Hepatol.* **31**, 78–83.

Alvaro, D., Cho, W.K., Mennone, A. and Boyer, J.L. (1993) "Effect of secretion on intracellular pH regulation in isolated rat bile duct epithelial cells", *J. Clin. Investig.* **92**, 1314–1325.

Alvaro, D., Gigliozzi, A., Fraioli, F., *et al.* (1997a) "Hormonal regulation of bicarbonate secretion in the biliary epithelium", *Yale J. Biol. Med.* **70**, 417–426.

Alvaro, D., Mennone, A. and Boyer, J.L. (1997b) "Role of kinases and phosphatases in the regulation of fluid secretion and $Cl^-/HCO_3^-$ exchange in cholangiocytes", *Am. J. Physiol.* **273**, G303–G313.

Alvaro, D., Alpini, G., Jezequel, A.M., *et al.* (1997c) "Role and mechanisms of action of acetylcholine in the regulation of rat cholangiocyte secretory functions", *J. Clin. Investig.* **100**, 1349–1362.

Ayres, R.C., Neuberger, J.M., Shaw, J., *et al.* (1993) "Intercellular adhesion molecule-1 and MHC antigens on human intrahepatic bile duct cells: effect of pro-inflammatory cytokines", *Gut* **34**, 1245–1249.

Bour, E.S., Ward, L.K., Cornman, G.A. and Isom, H.C. (1996) "Tumor necrosis factor-alpha-induced apoptosis in hepatocytes in long-term culture", *Am. J. Pathol.* **148**, 485–495.

Brandtzaeg, P. and Prydz, H. (1984) "Direct evidence for an integrated function of J. chain and secretory component in epithelial transport of immunoglobulins", *Nature* **311**, 71–73.

Caligiuri, A., Glaser, S., Rodgers, R.E., *et al.* (1998) "Endothelin-1 inhibits secretin-stimulated ductal secretion by interacting with ETA receptors on large cholangiocytes", *Am. J. Physiol.* **275**, G835–G846.

Cho, W.K., Mennone, A., Rydberg, S.A. and Boyer, J.L. (1997a) "Bombesin stimulates bicarbonate secretion from rat cholangiocytes: implications for neural regulation of bile secretion", *Gastroenterology* **113**, 311–321.

Cho, W.K., Mennone, A. and Boyer, J.L. (1997b) "VIP stimulates bile secretion in cholangiocytes by cAMP-PKA independent mechanisms", *Hepatology*, 26.

Cruickshank, S.M., Southgate, J., Selby, P.J. and Trejdosiewicz, L.K. (1998) "Expression and cytokine regulation of immune recognition elements by normal human biliary epithelial and established liver cell lines *in vitro*", *J. Hepatol.* **29**, 550–558.

Daniels, C.K. and Schmucker, D.L. (1987) "Secretory component-dependent binding of immunoglobulin A in the rat, monkey and human: a comparison of intestine and liver", *Hepatology* **7**, 517–521.

Daniels, C.K., Schmucker, D.L. and Jones, A.L. (1985) "Age-dependent loss of dimeric immunoglobulin A receptors in the liver of the Fischer 344 rat", *J. Immunol.* **134**, 3855–3858.

Delacroix, D.L., Courtoy, P.J., Rahier, J., *et al.* (1984) "Localization and serum concentration of secretory component during massive necrosis of human liver", *Gastroenterology* **86**, 521–531.

Eisenmann-Tappe, I., Wizigmann, S. and Gebhardt, R. (1991) "Glutamate uptake in primary cultures of biliary epithelial cells from normal rat liver", *Cell Biol. Toxicol.* **7**, 315–325.

Elsing, C., Kassner, A. and Stremmel, W. (1996) "Sodium, hydrogen antiporter activation by extracellular adenosine triphosphate in biliary epithelial cells", *Gastroenterology* **111**, 1321–1332.

Fitz, J.G. (1997) "Evidence for paracrine regulation of biliary $Cl^-$ secretion by purinergic signalling", In: Alvaro, D., Benedetti, A. and Strazzabosco, M., eds, Vanishing Bile Duct Syndrome—Pathophysiology (Kluwer Academic Publishers, London), pp 65–71.

Frizzel, R.A. and Morris, A.P. (1994) "Chloride conductance of salt-secreting epithelial cells", Current Topics in Membranes (Academic Press, New York) Vol. 42, pp 173–214.

Glaser, S.S., Rodgers, R.E., Phinizy, J.L., *et al.* (1997) "Gastrin inhibits secretin-induced ductal secretion by interaction with specific receptors on rat cholangiocytes", *Am. J. Physiol.* **273**, G1061–G1070.

Goldsmith, M.A., Jones, A.L., Underdown, B.J. and Schiff, J.M. (1987) "Alterations in protein transport events in rat liver after estrogen treatment", *Am. J. Physiol.* **253**, G195–G200.

Hahn, W.C., Burakoff, S.J. and Bierer, B.E. (1993) "Signal transduction pathways involved in T cell receptor-induced regulation of CD2 avidity for CD58", *J. Immunol.* **150**, 2607–2619.

Hanly, W.C., Cook, L., Kingzette, M., *et al.* (1987) "Rabbit secretory components: identification of a third allotype, t63", *J. Immunol.* **139**, 1597–1601.

Hansen, P.G., Hennessy, E.J., Blake, H., *et al.* (1989) "Appearance of IgG and IgA antibodies in human bile after tetanus toxoid immunization", *Clin. Exp. Immunol.* **77**, 215–220.

Harmatz, P.R., Kleinman, R.E., Bunnell, B.W., *et al.* (1982) "Hepatobiliary clearance of IgA immune complexes formed in the circulation", *Hepatology* **2**, 328–333.

Himeno, H., Saibara, T., Onishi, S., *et al.* (1992) "Administration of interleukin-2 induces major histocompatibility complex class II expression on the biliary epithelial cells, possibly through endogenous interferon-gamma production", *Hepatology* **16**, 409–417.

Hofmann, A.F., Z., Y.H., Schteingart, C.D., *et al.* (1997) "The cholehepatic circulation of organic anions: a decade of progress", In: Alvaro, D., Benedetti, A. and Strazzabosco, M., eds, Vanishing Bile Duct Syndrome—Pathophysiology (Kluwer Academic Publishers, London), pp 90–106.

Hoppe, C.A., Connolly, T.P. and Hubbard, A.L. (1985) "Transcellular transport of polymeric IgA in the rat hepatocyte: biochemical and morphological characterization of the transport pathway", *J. Cell Biol.* **101**, 2113–2123.

Jackson, G.D. and Walker, P.G. (1983) "The transient appearance of IgM antibodies in the bile of rats injected with Salmonella enteritidis", *Immunol. Lett.* **7**, 41–45.

Kloppel, T.M., Hoops, T.C., Gaskin, D. and Le, M. (1987) "Uncoupling of the secretory pathways for IgA and secretory component by cholestasis", *Am. J. Physiol.* **253**, G232–G240.

Kvale, D., Lovhaug, D., Sollid, L.M. and Brandtzaeg, P. (1988) "Tumor necrosis factor-alpha up-regulates expression of secretory component, the epithelial receptor for polymeric Ig", *J. Immunol.* **140**, 3086–3089.

Lazaridis, K.N., Pham, L., Tietz, P., *et al.* (1997) "Rat cholangiocytes absorb bile acids at their apical domain via the ileal sodium-dependent bile acid transporter", *J. Clin. Investig.* **100**, 2714–2721.

Lemaitre-Coelho, I., Jackson, G.D. and Vaerman, J.P. (1977) "Rat bile as a convenient source of secretory IgA and free secretory component", *Eur. J. Immunol.* **7**, 588–590.

Leon, M.P., Bassendine, M.F., Gibbs, P., *et al.* (1997) "Immunogenicity of biliary epithelium: study of the adhesive interaction with lymphocytes", *Gastroenterology* **112**, 968–977.

Lindh, E. and Bjork, I. (1976) "Binding of secretory component to dimers of immunoglobulin A *in vitro*. Mechanism of the covalent bond formation", *Eur. J. Biochem.* **62**, 263–270.

Lira, M., Schteingart, C.D., Steinbach, J.H., *et al.* (1992) "Sugar absorption by the biliary ductular epithelium of the rat: evidence for two transport systems", *Gastroenterology* **102**, 563–571.

Manning, R.J., Walker, P.G., Carter, L., *et al.* (1984) "Studies on the origins of biliary immunoglobulins in rats", *Gastroenterology* **87**, 173–179.

Martinez-Anso, E., Castillo, J.E., Diez, J., *et al.* (1994) "Immunohistochemical detection of chloride/bicarbonate anion exchangers in human liver", *Hepatology* **19**, 1400–1406.

Matsumoto, K., Fujii, H., Michalopoulos, G., *et al.* (1994) "Human biliary epithelial cells secrete and respond to cytokines and hepatocyte growth factors *in vitro*: interleukin-6, hepatocyte growth factor and epidermal growth factor promote DNA synthesis *in vitro*", *Hepatology* **20**, 376–382.

Mazanec, M.B., Kaetzel, C.S., Lamm, M.E., *et al.* (1995) "Intracellular neutralization of Sendai and influenza viruses by IgA monoclonal antibodies", *Adv. Exp. Med. Biol.* **371A**, 651–654.

Milani, S., Herbst, H., Schuppan, D., *et al.* (1991) "Transforming growth factors beta 1 and beta 2 are differentially expressed in fibrotic liver disease", *Am. J. Pathol.* **139**, 1221–1229.

Morland, C.M., Fear, J., McNab, G., *et al.* (1997) "Promotion of leukocyte transendothelial cell migration by chemokines derived from human biliary epithelial cells *in vitro*", *Proc. Assoc. Am. Physicians* **109**, 372–382.

Mullock, B.M., Jones, R.S., Peppard, J. and Hinton, R.H. (1980) "Effect of colchicine on the transfer of IgA across hepatocytes into bile in isolated perfused rat livers", *FEBS Lett.* **120**, 278–282.

Nagura, H., Nakane, P.K. and Brown, W.R. (1978) "Breast milk IgA binds to jejunal epithelium in suckling rats", *J. Immunol.* **120**, 1333–1339.

Nathanson, M.H. and Boyer, J.L. (1991) "Mechanisms and regulation of bile secretion", *Hepatology* **14**, 551–566.

Ogra, S.S., Ogra, P.L., Lippes, J. and Tomasi, T.B., Jr. (1972) "Immunohistologic localization of immunoglobulins, secretory component, and lactoferrin in the developing human fetus", *Proc. Soc. Exp. Biol. Med.* **139**, 570–574.

Pardo, A.G., Lamm, M.E., Plaut, A.G. and Frangione, B. (1979) "Secretory component is convalently bound to a single sub-unit in human secretory IgA", *Mol. Immunol.* **16**, 477–482.

Peppard, J.V., Orlans, E., Andrew, E. and Payne, A.W. (1982) "Elimination into bile of circulating antigen by endogenous IgA antibody in rats", *Immunology* **45**, 467–472.

Rao, C.K., Kaetzel, C.S. and Lamm, M.E. (1987) "Induction of secretory component synthesis in colonic epithelial cells", *Adv. Exp. Med. Biol.* **216B**, 1071–1077.

Schiff, J.M., Fisher, M.M., Jones, A.L. and Underdown, B.J. (1986) "Human IgA as a heterovalent ligand: switching from the asialoglycoprotein receptor to secretory component during transport across the rat hepatocyte", *J. Cell Biol.* **102**, 920–931.

Scholz, M., Cinatl, J., Blaheta, R.A., *et al.* (1997) "Expression of human leukocyte antigens class I and class II on cultured biliary epithelial cells after cytomegalovirus infection", *Tissue Antigens* **49**, 640–643.

Singh, S.K., Mennone, A. and Boyer, J.L. (1996) "$Na^+$–$K^+$–$2Cl^-$ cotransport participates in stimulated fluid secretion in isolated polarized bile duct units from rat liver", *Hepatology* **26**(279A), (Abstract).

Socken, D.J. and Underdown, B.J. (1978) "Comparison of human, bovine and rabbit secretory component-immunoglobulin interactions", *Immunochemistry* **15**, 499–506.

Socken, D.J., Simms, E.S., Nagy, B.R., *et al.* (1981) "Secretory component-dependent hepatic transport of IgA antibody–antigen complexes", *J. Immunol.* **127**, 316–319.

Solari, R., Racine, L., Tallichet, C. and Kraehenbuhl, J.P. (1986) "Distribution and processing of the polymeric immunoglobulin receptor in the rat hepatocyte: morphological and biochemical characterization of subcellular fractions", *J. Histochem. Cytochem.* **34**, 17–23.

Sollid, L.M., Kvale, D., Brandtzaeg, P., *et al.* (1987) "Interferon-gamma enhances expression of secretory component, the epithelial receptor for polymeric immunoglobulins", *J. Immunol.* **138**, 4303–4306.

Spirli, C., Granato, A., Zsembery, K., *et al.* (1998) "Functional polarity of $Na^+/H^+$ and $Cl^-/HCO_3^-$ exchangers in a rat cholangiocyte cell line", *Am. J. Physiol.* **275**, G1236–G1245.

Strazzabosco, M. (1997) "New insights into cholangiocyte physiology", *J. Hepatol.* **27**, 945–952.

Strazzabosco, M., Joplin, R., Zsembery, A., *et al.* (1997) "Na(+)-dependent and -independent $Cl^-/HCO_3^-$ exchange mediate cellular $HCO_3^-$ transport in cultured human intrahepatic bile duct cells", *Hepatology* **25**, 976–985.

Strazzabosco, M., Spirli, C. and Okolicsanyi, L. (2000) "Pathophysiology of the intrahepatic biliary epithelium", *J. Gastroenterol. Hepatol.* **15**, 244–253.

Sullivan, D.A. and Wira, C.R. (1981) "Estradiol regulation of secretory component in the female reproductive tract", *J. Steroid Biochem.* **15**, 439–444.

Sullivan, D.A., Bloch, K.J. and Allansmith, M.R. (1984) "Hormonal influence on the secretory immune system of the eye: androgen regulation of secretory component levels in rat tears", *J. Immunol.* **132**, 1130–1135.

Takahashi, I., Nakane, P.K. and Brown, W.R. (1982) "Ultrastructural events in the translocation of polymeric IgA by rat hepatocytes", *J. Immunol.* **128**, 1181–1187.

Tavoloni, N. (1987) "The intrahepatic biliary epithelium: an area of growing interest in hepatology", *Semin. Liver Dis.* **7**, 280–292.

Tietz, P.S., Alpini, G., Pham, L.D. and Larusso, N.F. (1995) "Somatostatin inhibits secretin-induced ductal hypercholeresis and exocytosis by cholangiocytes", *Am. J. Physiol.* **269**, G110–G118.

Vos, T.A., Gouw, A.S., Klok, P.A., *et al.* (1997) "Differential effects of nitric oxide synthase inhibitors on endotoxin-induced liver damage in rats", *Gastroenterology* **113**, 1323–1333.

Wira, C.R. and Colby, E.M. (1985) "Regulation of secretory component by glucocorticoids in primary cultures of rat hepatocytes", *J. Immunol.* **134**, 1744–1748.

Yasoshima, M., Kono, N., Sugawara, H., *et al.* (1998) "Increased expression of interleukin-6 and tumor necrosis factor-alpha in pathologic biliary epithelial cells: *in situ* and culture study", *Lab. Investig.* **78**, 89–100.

